# Radio Frequency Interference Suppression for High-Frequency Ocean Remote Sensing Radar with Inter-Pulse Phase Agility Waveform

**DOI:** 10.3390/s26082350

**Published:** 2026-04-10

**Authors:** Heng Zhou, Xiongbin Wu, Liang Yu, Fuqi Mo, Xiaoyan Li

**Affiliations:** School of Earth and Space Science and Technology, Wuhan University, Wuhan 430072, China; hzhou@whu.edu.cn (H.Z.); liang_yu@whu.edu.cn (L.Y.); mfq2014@whu.edu.cn (F.M.); lixiaoyan@whu.edu.cn (X.L.)

**Keywords:** HFSWR, radio interference, inter-pulse phase agility, orthogonal projection

## Abstract

The inversion of wind and wave parameters in high-frequency ocean remote sensing radar relies heavily on the sea echo Doppler power spectrum. However, the accuracy of parameter inversion is often compromised by radio frequency interference (RFI), which distorts the Doppler spectral power distribution. Existing RFI suppression algorithms primarily focus on enhancing the signal-to-interference-plus-noise ratio post-mitigation, while insufficient attention has been paid to the spectral power fluctuations induced by these suppression processes. To address this issue, this study proposes a narrowband RFI suppression scheme that combines inter-pulse phase agility (IPA) with orthogonal projection (OP). An optimized aperiodic sequence is used to modulate the inter-pulse phases of the transmitted waveform, thus uniformly dispersing the sea echo power across the entire Doppler spectrum. Spatial OP is then applied to suppress RFI stripes on the range-Doppler spectrum, a process in which only the sea echo samples masked by the RFI stripes are affected. Finally, phase compensation restores the sea echo coherence and disperses residual RFI power uniformly into the Doppler domain, minimizing its localized impact. Simulations and semi-synthetic tests involving real-world interference verify that the proposed scheme effectively suppresses RFI while alleviating spectral distortion in the sea-echo Doppler spectrum.

## 1. Introduction

High-frequency surface wave radar (HFSWR) serves as a robust instrument for maritime target detection [1,2,3] and the characterization of ocean dynamic parameters [4,5,6,7]. It facilitates the wide-area sensing of surface currents, winds, and waves over expansive maritime regions. Based on Barrick’s first- and second-order scattering theories, ocean dynamics parameters can be extracted from the Doppler spectrum of sea echoes. Specifically, wind direction is estimated from the power ratio of the first-order positive and negative Bragg peak [8], a process that is highly sensitive to the power levels of Bragg peaks. Wave and wind parameters are retrieved from the Doppler power distribution of both first- and second-order sea echoes [9,10]. However, in the increasingly congested high frequency (HF) band, the Doppler power spectrum is often severely contaminated by external interference, which significantly degrades the accuracy of sea-state parameter inversion [11]. This is particularly critical for narrowband radio frequency interference (RFI), which manifests as high-intensity stripes in the Doppler domain. Owing to the intense power of the RFI, its signal-to-noise ratio (SNR) exceeds the suppression capability of the beamformer’s sidelobes, which are insufficient to prevent contamination of the relatively weak sea echoes in adjacent azimuth channels. Effective RFI suppression is an essential precursor to parameter inversion. And it is imperative that the mitigation process minimizes any unintended distortion to the Doppler power distribution of the sea echoes.

Various signal processing methods have been developed to mitigate RFI by exploiting the distinct characteristics of interference across different domains. Spatial adaptive beamforming is a conventional technique widely employed in skywave radar systems [12,13], which leverages the directional characteristics of RFI to achieve suppression by steering adaptive nulls toward the interference sources. However, conventional adaptive beamforming often results in inevitable main-lobe signal cancelation when the desired signals overlap with the interference within the main lobe. This issue is particularly pronounced in HF ocean radars, which typically employ small-aperture arrays or compact monopole/cross-loop antennas characterized by highly constrained spatial degrees of freedom. Significant efforts have been directed toward employing spatial orthogonal projection (OP) algorithms as an alternative to conventional adaptive beamforming [14,15]. Analogous to adaptive beamforming, spatial OP methods necessitate interference-only data blocks to estimate the spatial interference covariance matrix and its associated subspace eigenvectors. Given that the eigenvectors and the interference steering vectors span the same spatial subspace, spatial OP inherently attenuates sea echoes co-located within the interference main lobe.

The distinct characteristics of RFI and sea echoes in the slow-time domain (i.e., the time scale across successive pulses within a coherent integration period) have also been exploited for interference separation and mitigation. For short-duration RFI, which typically exhibits significantly higher amplitudes than sea echoes, corrupted samples are generally identified and replaced by reconstructed data synthesized via techniques such as linear prediction [16], compressive sensing [17], empirical mode decomposition [18], and singular value decomposition (SVD). However, the performance of these reconstruction-based methods is often limited by reconstruction errors. As the number of RFI-contaminated sweeps increases, the expanding volume of samples requiring reconstruction inevitably leads to greater distortions in the Doppler spectrum power distribution of sea echoes. Furthermore, OP filtering has been applied in the slow-time domain for RFI suppression [19]. Given that narrowband interference is typically distributed across all range bins and exhibits strong cross-range correlation [20], the interference subspace can be constructed using slow-time sequences from reserved range bins which contain only interference. For narrowband RFI which manifests as stripes parallel to the range axis on the range-Doppler spectrum (RDS), its consistent spectral structure across different range bins allows for RFI mitigation via frequency-domain OP [21].

To minimize the collateral degradation of the sea echo Doppler spectrum during RFI mitigation, research can be directed toward two primary objectives. First, it is essential to develop suppression algorithms to achieve superior separability between RFI and sea echoes. Second, selecting the optimal processing domain is critical to reducing the ‘mitigation footprint’ (i.e., the volume of processed samples). For instance, with narrowband RFI that persists throughout the coherent integration time (CIT)—defined as the temporal window over which the received echoes are processed using a Fourier transform to achieve Doppler separation—time-domain suppression would inevitably compromise the entire data record. In contrast, shifting the mitigation to the Doppler domain confines the processing impact to only the localized bins masked by RFI stripes, thereby preserving the spectral integrity of the remaining Doppler cells.

Inter-pulse waveform agility serves as a pivotal feature of modern cognitive radar systems. This technique enables the pseudo-random optimization of the key modulation parameters—including amplitude, phase, emission time offset, and carrier frequency—across pulse sequences [22]. While extensively deployed for clutter suppression [23,24] and the mitigation of signal-dependent jamming [25,26,27], waveform agility also holds significant potential for countering signal-independent narrowband RFI. Since RFI originates from external radiation sources, the use of an inter-pulse phase agility (IPA) waveform does not modify the RFI component present in the received data. In contrast, the phase of all backscattered signals—such as target echoes, sea clutter, and ionospheric returns—is governed by the transmit phase modulation. By applying a pre-defined pseudo-random phase modulation, these desired signals and active clutter are uniformly spread across all Doppler bins, whereas the narrowband RFI stripes remain concentrated and unaffected. Suppressing RFI within this specific RDS significantly reduces the attenuation of desired signals typically caused by interference mitigation, thereby preserving the spectral integrity of the Doppler spectrum.

The main contribution of this research is the development of a narrowband RFI suppression scheme synergistically combines IPA waveform design with spatial orthogonal projection. The synergy between the IPA waveform and localized Doppler-domain processing effectively alleviates the signal self-nulling typically encountered in traditional spatial filtering when addressing narrowband strip-like RFI when addressing narrowband strip-like RFI. Validation using semi-synthetic datasets demonstrates that the technique effectively suppresses RFI while maintaining the average power deviation of ocean echoes within a 1 dB margin. Consequently, this methodology shows significant potential for enhancing both the operational stability and the sea-state inversion accuracy of HFSWR systems in spectrally congested environments.

The remainder of this paper is structured as follows. Section 2 establishes the theoretical principles of the IPA waveform and elaborates on the proposed interference suppression pipeline, including the methodology for phase sequence optimization and the detection of RFI stripes. Section 3 evaluates the performance of the framework through simulations and semi-synthetic data experiments. The generalization performance of the proposed scheme is discussed in Section 4, and Section 5 concludes the paper with final remarks.

## 2. Materials and Methods

### 2.1. Signal Model of IPA Waveform

HFSWR systems designed for ocean remote sensing commonly employ linear frequency modulated interrupted continuous wave (FMICW) as the transmit waveform. By performing dechirping for range compression, the system effectively reduces both the sampling rate requirements and the overall hardware costs. For analytical tractability, the FMICW radar signal is typically modeled as a conventional linear frequency modulated continuous wave (FMCW) waveform. When a radar employs a conventional FMCW waveform, the transmitted signal during a single sweep period can be expressed as follows:

(1) $$s_{t}\left(t\right)=\exp\left[j2\pi \left(f_{c}t+\frac{1}{2}kt^{2}\right)\right],\;\;0<t<T$$

where $f_{c}$ denotes the carrier frequency, k is the chirp rate, and T represents the sweep duration. The initial phase of $s_{t}$ is omitted since it remains constant across successive sweeps in a conventional FMCW waveform. When the FMCW waveform is modulated with an inter-pulse phase agile sequence, the transmitted signal for the n-th sweep period can be expressed as:

(2) $${\tilde{s}}_{t}\left(t,n\right)=\exp\left[j\left(2\pi f_{c}t+\pi kt^{2}+\varphi_{n}\right)\right],\;\;0<t<T$$

where $\varphi_{n}$ denotes the agile phase of the n-th sweep. Unlike conventional FMCW waveform, the initial phases of the IPA waveform vary across sweep periods according to a predefined sequence. After mixing with the signal defined in Equation (1), range-compressed samples of the IPA waveform retain the same inter-pulse phase modulation as the original transmitter. The beat signal of IPA waveform for the n-th sweep period can be formulated as:

(3) $${\tilde{s}}_{b}\left(t,n\right)\approx A\exp\left[j\left(2\pi \left(\frac{2kR}{c}\right)t+2\pi f_{d}nT+\varphi_{n}+\varphi_{0}\right)\right]$$

where A is the amplitude of the beat signal depending on target radar cross section, path loss and receiver gain, R, $f_{d}$ and c represent the target range for the first sweep, target Doppler frequency and light speed, respectively, $\varphi_{0}$ is the residual constant phase remaining after the dechirp process. By neglecting the phase terms that are invariant across sweeps, the simplified expression for the slow-time signal at the specific range bin where the target is located can be formulated as follows:

(4) $${\tilde{s}}_{b}\left(n\right)=A\exp\left[j\left(2\pi f_{d}nT+\varphi_{n}\right)\right]=A\cdot g\left(n\right)\cdot q\left(n\right),\;\;\;n=0,1,\cdots ,N-1$$

where $g\left(n\right)=exp(j2\pi f_{d}nT)$ denotes the Doppler phase term, $q\left(n\right)=exp(j\varphi_{n})$ denotes the inter-pulse phase agile sequence term. For a conventional transmit waveform, $\varphi_{n}=0$, the slow-time signal becomes:

(5) $$s_{b}\left(n\right)=A\exp\left(j2\pi f_{d}nT\right)=Ag\left(n\right),\;\;\;n=0,1,\cdots ,N-1$$

Let G(k) denote the discrete Fourier transform of g(n), where k(k=0,1,⋯,N−1) represents the discrete Doppler frequency index. For a coherent integration of N sweeps, the Doppler response of the target echo for conventional FMCW waveform can be expressed as:

(6) $$S_{b}\left(k\right)=AG\left(k\right),\;\;\;k=0,1,\cdots ,N-1$$

And the Doppler response for IPA-FMCW waveform can be expressed as:

(7) $${\tilde{S}}_{b}\left(k\right)={\frac{1}{N}S}_{b}\left(k\right)*Q\left(k\right)=\frac{A}{N}G\left(k\right)*Q\left(k\right),\;\;\;k=0,1,\cdots ,N-1$$

where symbol ∗ denotes the convolution operator. Q(k) denotes the discrete Fourier transform of q(n).

Equation (7) shows that the Doppler spectrum with IPA-FMCW waveform is the convolution of the conventional Doppler spectrum and the spectrum of the phase modulation sequence. If the inter-pulse phase agile sequence is a random sequence uniformly distributed within [0, 2π), its spectrum will exhibit white-noise-like characteristics. In this case, the echo power is approximately uniformly dispersed across all Doppler bins. A phase compensation procedure based on the predefined phase sequence must be performed to restore the coherence of the echo signals.

### 2.2. Impact of IPA Waveform on RFI

Narrowband RFI is a significant factor limiting the performance of HFSWR. In the radar receiver, narrowband RFI is typically modeled as a superposition of a series of single-frequency continuous wave signals in the fast-time domain, which refers to the sampling interval within a single sweep that corresponds to the range dimension. After de-chirping, the narrowband RFI is transformed into a swept-frequency signal with a modulation slope opposite to that of the transmitted signal. Consequently, the Range-FFT (fast Fourier transform) process spreads the RFI energy across all range bins. For a single narrowband RFI source, the interference maintains high coherence in the slow-time domain; thus, after Doppler processing, it manifests as a high-power stripe parallel to the range axis, with its energy concentrated within a few specific Doppler bins. Consequently, at any given range bin, the slow-time narrowband RFI can be modeled as a superposition of multiple single-frequency signals.

Assuming a single-tone interference with the same Doppler as the target exists at the corresponding range bin, the slow-time signal for a conventional waveform can be expressed as:

(8) $$x(n)=s_{b}(n)+s_{j}(n)+w(n)$$

where w(n) represents the additive white Gaussian noise in the slow-time, $s_{j}(n)$ is a single-tone interference:

(9) $$s_{j}(n)=A_{j}\exp\left(j2\pi f_{d}nT\right)$$

where $A_{j}$ is the complex amplitude of the single-frequency interference component. The Doppler spectrum of the RFI overlaps with that of the target, leading to a degradation of the signal-to-interference-plus-noise ratio (SINR) at the target-occupied Doppler bins. When the IPA-FMCW waveform is employed, the slow-time signal can be expressed as:

(10) $$\tilde{x}\left(n\right)=s_{b}\left(n\right)\cdot q(n)+s_{j}(n)+w(n)$$

After phase compensation, $\tilde{x}\left(n\right)$ is transformed into $\tilde{y}\left(n\right)$ as follows:

(11) $$\tilde{y}\left(n\right)=s_{b}\left(n\right)+s_{j}(n)\cdot q^{*}(n)+w(n)\cdot q^{*}(n)$$

where ${\left[\cdot \right]}^{*}$ denotes the complex conjugate operation. After the Doppler FFT, the Doppler spectra of the conventional FMCW waveform and the IPA-FMCW waveform prior to phase compensation can be respectively expressed as X(k) and $\tilde{X}(k)$, which are given by:

(12) $$X\left(k\right)=AG\left(k\right)+A_{j}G\left(k\right)+W(k)$$

(13) $$\tilde{X}\left(k\right)=\frac{A}{N}G\left(k\right)*Q(k)+A_{j}G\left(k\right)+W(k)$$

where W(k) denotes the additive white noise spectrum after Doppler-FFT.

Comparing Equation (8) with Equation (10), it is evident that the IPA waveform disrupts the phase coherence of the target echoes. When the phase-agile sequence follows a uniform random distribution, it exhibits a white-noise-like spectrum, which disperses the target signal power across all Doppler bins without affecting external RFI sources. By performing localized suppression on the RFI stripes in the RDS, only a small fraction of the target samples overlapping with the interference is affected. This significantly reduces the total desired signal power loss caused by the interference suppression process.

According to Equation (13), the desired signal power contained in each Doppler cell of the IPA-FMCW echo RDS is only 1/N of the total power. Assuming that the RFI occupies $N_{J}$ Doppler bins and the desired signal components within these bins are completely suppressed during interference mitigation, the target power loss (in dB) after restoring the echo coherence can be expressed as:

(14) $$L_{IPA}=-10\;\log_{10}\left(1-\frac{N_{j}}{N}\right)$$

Since the power of narrowband RFI is typically concentrated within a limited number of Doppler bins (i.e., $N_{j}\ll N$), the resulting value of the power loss $L_{IPA}$ remains negligible. After phase compensation, the resulting representation of the IPA waveform in the Doppler domain is given by:

(15) $$\tilde{Y}\left(k\right)=AG\left(k\right)+\frac{A_{j}}{N}G\left(k\right)*Q^{*}(N-k)+\frac{1}{N}W(k)*Q^{*}(N-k)$$

While restoring the coherence of the desired signal, the RFI is decorrelated by the phase compensation process, which spreads its power across the entire Doppler domain. Assuming the average residual RFI power across $N_{J}$ interference Doppler bins after suppression is $P_{J}$, the signal-to-interference ratio (SIR) at the target Doppler bin for a conventional waveform is given by:

(16) $${SIR}_{conv}=\frac{{\left|A\right|}^{2}}{P_{J}}$$

While the SIR at the target Doppler bin for IPA waveform is expressed as:

(17) $${SIR}_{IPA}=\frac{N}{N_{J}}\cdot \frac{{\left|A\right|}^{2}}{P_{J}}=\frac{N}{N_{J}}{SIR}_{conv}$$

Compared to the conventional waveform, the phase compensation process of the IPA waveform further suppresses the peak power of the residual RFI in the Doppler spectrum. This prevents the formation of false spectral peaks caused by residual interference, while simultaneously minimizing its impact on the Doppler power spectrum of the sea echo.

### 2.3. Optimization of Inter-Pulse Phase Agile Sequence

The IPA waveform ensures that either the echo signal or the external RFI—but never both—maintains coherence, thereby mitigating the impact of interference suppression and residual RFI on the echo power. While an independent and identically distributed random phase sequence achieves ideal decorrelation in the limit, a finite-length sequence inevitably exhibits spectral ripples. This spectral non-uniformity causes RFI energy to cluster in specific Doppler bins, creating residual spikes that increase the power fluctuations of the sea echoes after interference mitigation. Therefore, optimizing the spectral flatness of the phase-agile sequence is essential for maximizing suppression efficacy and the signal-to-interference-plus-noise ratio.

The objective of the inter-pulse phase-agile sequence design is to achieve a power spectral density that is as uniform as possible. According to the Wiener–Khinchin theorem, optimizing spectral flatness is mathematically equivalent to driving the autocorrelation of the phase sequence toward zero for all non-zero time lags. Consequently, the inter-pulse phase-agile sequence design is formulated as an aperiodic sequence optimization problem under the peak sidelobe level (PSL) criterion. To synthesize a sequence characterized by a ‘thumbtack-like’ aperiodic autocorrelation function, the optimization task is formulated as follows:

(18) min φ1,⋯φNmax k≠0∑n=1N−ksnsn+k*s. t.    sn=ejφn,   φ∈0,2π,   n=1,…N

Design methodologies for aperiodic sequences have been extensively investigated [28], with the alternating projection method being one well-established frameworks. In this approach, the sequence is designed by iteratively projecting the candidate sequence between two distinct constraint sets: the structural constraint set (e.g., constant modulus) and the aperiodic correlation performance set (e.g., minimal PSL). During each iteration, one subset of variables is optimized while the rest are held constant. This process continues until the performance metric converges or the maximum iteration limit is reached. A detailed implementation of this alternating projection method is provided in [28].

### 2.4. Proposed Narrowband RFI Mitigation Scheme

The IPA waveform redistributes the power of echo signals and external RFI in the RDS, preventing the desired signal from clustering in specific Doppler bins where it could be completely masked by strong interference. This redistribution significantly reduces the power loss of the desired signal during the interference suppression process. The advantages of the IPA waveform are realized only when combined with Doppler-domain suppression techniques. Currently, prominent Doppler-domain cancelation methods include adaptive beamforming and frequency-domain orthogonal projection. However, spatial filtering is rarely applied in HF ocean remote sensing radar, as it causes severe attenuation of sea echoes within the interference main lobe, thereby degrading the accuracy of sea state parameter inversion in those regions.

**Figure 1:**
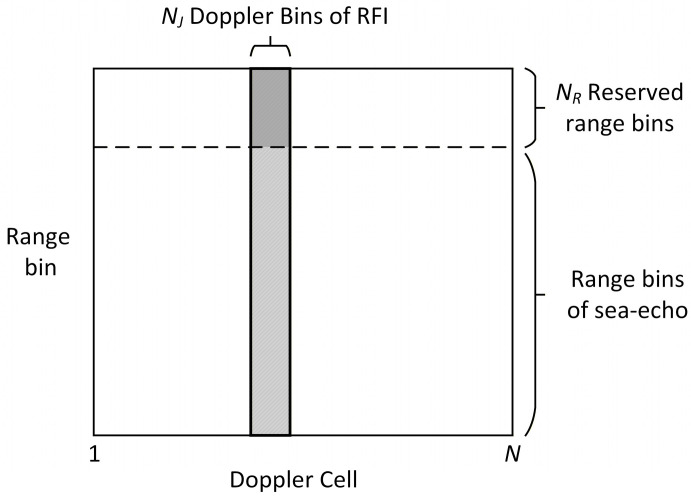
Diagrammatic illustrations of the spatial orthogonal projection in Doppler domain.

According to Equation (14), the introduction of the IPA waveform can substantially mitigate the signal power loss caused by localized spectral processing. In this study, a spatial orthogonal projection algorithm is employed to suppress interference stripes in the Doppler domain before phase compensation. Specifically, samples from preserved range bins within the interference-contaminated Doppler range (indicated by the gray shaded area in Figure 1) are utilized to estimate the spatial covariance matrix of the RFI. Subsequently, the interference subspace is constructed to perform orthogonal projection processing on the sea-echo range bins affected by the RFI stripes.

The processing steps of proposed scheme are as follows:

(1) Calculation of the multi-channel echo range-Doppler spectrum;
(2) Identify RFI-contaminated Doppler bins and determine those requiring OP processing by evaluating the interference-to-noise ratio (INR) within the interference-only range bins of the RDS.
(3) Construct the interference spatial covariance matrix ${\hat{R}}_{x}$ using samples from the reserved range bins within the RFI-contaminated Doppler cells.
  (19) $${\hat{R}}_{x}=\frac{1}{N_{J}N_{R}}XX^{H}$$
  where $X={\left[x_{1},\;x_{2},\;\ldots x_{M}\right]}^{T}$, M is the number of receiving channels. ${\left[\cdot \right]}^{H}$ denotes the conjugate transpose. $x_{m}$ denotes the $N_{J}N_{R}\times 1$ vector consisting of interference samples collected from the m-th channel, spanning the interference-contaminated Doppler bins and the reserved range bins.
(4) Estimation of the interference subspace via SVD:
  (20) $${\hat{R}}_{x}=U\Sigma V^{H}$$
  where S and V denote the left and right singular vectors. Σ is a diagonal matrix containing the M singular values $\sigma_{1}\geq \sigma_{2}\geq \cdots \geq \sigma_{M}$, in which the largest singular values correspond to the dominant RFI components. The interference subspace is given by:
  (21) $$U_{J}=U(:,1:J)$$
  where J denotes the number of singular values associated with the interference. J is estimated using the second-order statistic of eigenvalues method [29].
(5) Spatial OP with
  (22) $$\tilde{y}=(I-U_{J}U_{J}^{H})y$$
  where I is the identity matrix. y denotes the spatial signal snapshot extracted from the RFI-contaminated Doppler bins within the specific sea-echo range cells of interest. $\tilde{y}$ represents the signal after spatial interference suppression. To achieve clean ocean-echo spectra, all RFI-contaminated Doppler bins are subjected to the OP.
(6) Transform the RFI-suppressed Doppler data back to the slow-time domain via an inverse FFT and perform the phase compensation to restore the sea-echo phase coherence.
(7) Conduct Doppler-FFT to obtain reconstructed sea-echo Doppler spectrum.

### 2.5. Identify the RFI-Contaminated Doppler Bins

In the preceding section, the detailed implementation steps of the proposed RFI suppression algorithm were presented. Among these, step (2) is critical as it dictates the number of Doppler bins subjected to OP. Processing an excessive number of RFI-contaminated Doppler bins inevitably leads to greater attenuation of the desired signal. Therefore, a reasonable INR threshold is essential to identify Doppler bins with concentrated interference power while simultaneously limiting the total number of bins designated for suppression. According to Equation (17), the phase compensation process suppresses RFI power by a factor of $N/N_{J}$. Consequently, even if certain RFI-affected bins are not suppressed due to an INR below the threshold, they will not significantly elevate the noise floor of the Doppler spectrum after phase compensation. In practice, a 3 dB INR threshold is generally sufficient to mitigate the majority of RFI power, resulting in a clean RDS.

The specific Doppler bins requiring RFI suppression are determined as follows:

(1) Noise floor estimation

Estimate the background noise floor level, denoted as $P_{noise}$, by utilizing designated regions of the RDS that are verified to be free of RFI and clutter.

(2) Average RFI spectrum calculation

Compute the average Doppler power spectrum, $P_{avg}(f_{d})$, by averaging the power across the interference-only range bins (typically selected from far-range cells where sea echoes are absent).

(3) Thresholding and identification

Define a detection threshold η=3 dB. A Doppler bin is identified as a candidate spectral point for RFI suppression only if it belongs to a cluster of consecutively appearing Doppler bins whose average power exceeds the noise floor by more than η (i.e., $P_{avg}\left(f_{d}\right)>P_{noise}+\eta$).

In Step (1), the noise floor is estimated by intentionally excluding high-power RFI strips. Given that the presence and Doppler occupancy of RFI are unknown a priori, a block-segmentation strategy is employed. The RDS is partitioned into multiple sub-blocks, each comprising 50–100 range-Doppler cells. A Gaussianity test is conducted on the real and imaginary components of each block, followed by an average power calculation. Data blocks that exhibit significant non-Gaussian behavior or an average power substantially exceeding the global median are discarded in their entirety from the estimation process. The noise floor is subsequently calculated using samples from all the remaining “clean” blocks. While this process effectively eliminates range bins dominated by strong ionospheric clutter, diffuse (blob-like) ionospheric clutter may not be fully excluded due to its distributed power profile, potentially introduces a slight positive bias into the noise floor estimation relative to the true ambient level.

In step (2), the Doppler power spectra from multiple interference-only range bins are averaged to estimate the RFI power profile. This approach leverages the fact that narrowband RFI strips on the RDS typically share a similar spectral signature across different range cells. Although the absolute RFI power may undergo significant variations between range bins, by performing ensemble averaging, the algorithm effectively captures the common spectral morphology of the interference.

In Step (3), a Doppler bin is identified as a candidate for suppression only if it constitutes part of a sequence of consecutive bins that exceed the predefined threshold. While a 3 dB INR threshold might appear relatively low, its efficacy is justified by the unique properties of the IPA waveform. Specifically, the phase agility causes the power of all intrinsic signals—including dominant ionospheric clutter and weaker ocean echoes—to be spread across the entire Doppler spectrum. Within this effectively “whitened” average Doppler profile, the 3 dB INR margin is sufficient to reliably distinguish RFI strips from the redistributed background. Furthermore, the continuity constraint serves as a vital safeguard, enabling the algorithm to differentiate genuine RFI signatures from isolated stochastic noise fluctuations or clutter spikes that may occasionally surpass the power threshold.

For Doppler bins with an INR below the detection threshold, the spatial OP algorithm is bypassed. However, during the phase compensation and coherent integration process—aimed at restoring signal coherency—these unsuppressed weak interference components are subjected to phase modulation (as defined in Equation (15)). Consequently, their power is dispersed across the entire Doppler dimension. This spectral whitening effect effectively reduces the average interference power per Range-Doppler cell, thereby preventing the formation of residual artifacts or false peaks that could otherwise impede target detection.

Notably, when RFI strips are absent from the RDS, the aforementioned detection procedure will not identify any candidate Doppler bins for suppression. In such scenarios, the spatial OP stage is entirely bypassed. Following the phase compensation process, the desired echo signals successfully restore their coherent integration gain. Since no OP processing is performed, the target signals suffer no attenuation. Furthermore, while the phase compensation restores the desired echoes, it simultaneously applies a random phase modulation to the asynchronous external noise. This induces a spectral whitening effect, where localized noise fluctuations are decorrelated and redistributed, ultimately leading to a more uniform power spectral density across the RDS.

### 2.6. Computational Complexity

Compared with the conventional 2D-FFT processing for Doppler estimation, the additional computational overhead of the proposed RFI suppression method primarily involves three components: the SVD for interference subspace extraction, the OP for interference nulling, and the inverse FFT/FFT operations required for phase compensation and coherent accumulation recovery. The total complexity can be broken down into three main stages, as summarized in Table 1.

While the total number of floating-point operations is dominated by the FFT pre-processing stage, the computational kernel of the proposed RFI suppression lies in the SVD with a complexity of $O\left(M^{3}\right)$. Owing to the relatively small number of antennas M typical in HFSWR systems, the proposed scheme remains highly efficient.

## 3. Results

### 3.1. Optimized Inter-Pulse Phase-Agile Sequence

**Figure 2:**
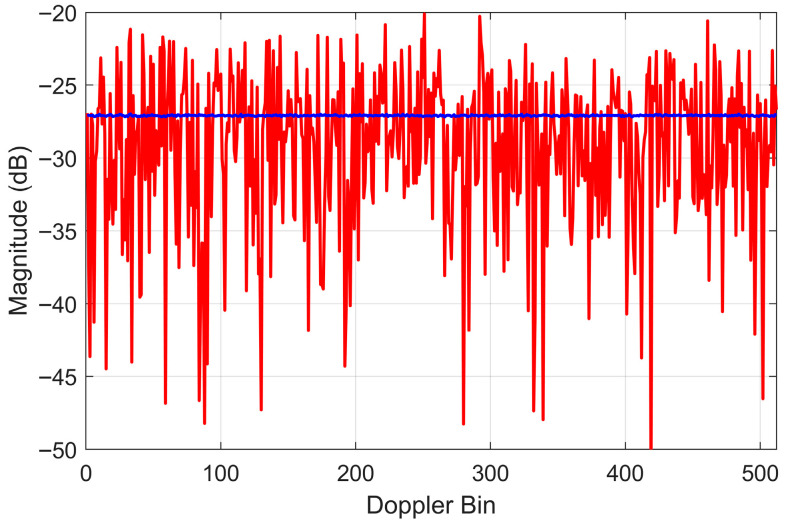
Doppler spectra of 512-length inter-pulse phase-agile sequences. The red line represents the power spectrum of a uniform random sequence (the initial sequence for the optimization). The blue line denotes the spectrum of the optimized sequence synthesized via the alternating projection method.

An aperiodic sequence with a minimized peak sidelobe level (PSL) effectively disperses the residual interference power across the entire Doppler domain. By distributing the spectral distribution of the RFI, the peak power density at any given spectral point is significantly reduced, thereby minimizing localized distortions to the sea-echo Doppler spectrum. Figure 2 illustrates the spectrum of a 512-point aperiodic sequence synthesized using the alternating projection method after 200 iterations. For comparison, the spectrum of an independent and identically distributed random phase sequence is also provided. For a random phase sequence, the standard deviation of spectral fluctuations is 5.4 dB, with a peak side lobe level of −20.1 dB. After optimizing with the alternating projection method, the standard deviation decreases significantly to 0.04 dB, and the peak level decreases to −27.0 dB, which is close to the theoretical processing gain limit of −27.1 dB (calculated as $-10log_{10}$512). Compared to the random phase sequence, the optimized sequence exhibits superior spectral flatness, which effectively ensures the uniform whitening of residual RFI and prevents the formation of structured interference artifacts in the Doppler domain.

### 3.2. Data Collection Setup

The proposed scheme can effectively alleviate the power fluctuations of sea echoes caused by interference suppression, primarily due to the implementation of the IPA waveform. This waveform alters the distribution characteristics of the desired echoes and external interference within the RDS. Ideally, the advantages of the IPA waveform should be validated by comparing it with a conventional waveform under identical sea states and RFI environments. However, such datasets are unattainable in field experiments because RFI observed using HFSWR systems is inherently time-varying—particularly its spectral footprint—and the dynamic nature of the sea surface prevents the acquisition of identical Doppler spectra across independent sampling intervals. To facilitate a controlled comparative study, this paper adopts a semi-synthetic approach. High-quality field-measured sea echoes, free from external interference and ionospheric clutter, are selected as the desired signals. IPA-encoded data are then generated by applying inter-pulse phase modulation to this desired signal. The external RFI components are synthesized through simulation and extracted from other field-measured RDS profiles. This methodology ensures that the desired signal and the interference background remain consistent for a comparative analysis across different waveforms.

**Figure 3:**
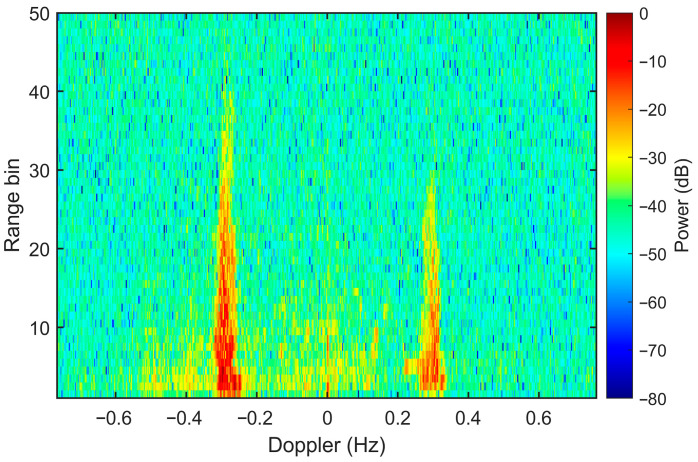
Interference free RDS from field-measured data with conventional waveform collected by OSMAR071 system on 12 September 2021, at 7:10 local time.

Figure 3 shows the RDS derived from field-measured data using a conventional FMICW waveform, with a range resolution of 5 km per bin and a coherent integration of 512 sweeps. This RDS is characterized by high signal-to-noise-ratio (SNR) sea echoes and the absence of external interference or ionospheric clutter. Consequently, this field-recorded dataset is employed as the desired sea-echo component for simulation validation. The data were collected by the ocean state monitoring and analyzing radar 071 (OSMAR071) system, an operational medium-range radar deployed on Wanshan Island (21.9° N, 113.7° E) in the South China Sea. The 8-element receive array comprises a 6-element uniform linear array (ULA) supplemented by two additional elements positioned in a second row. This array configuration satisfies the practical design constraints, maintaining a total aperture of less than 100 m at an operational frequency of 8 MHz. Crucially, it facilitates the effective attenuation of spatial pseudospectrum peaks induced by high-elevation (near-zenith, approaching 90°) ionospheric clutter Furthermore, the inclusion of nearly equilateral triangle sub-arrays enhances the estimation stability of channel amplitude and phase errors within the ocean-echo-based self-calibration algorithm [30]—the standard operational approach employed in the OSMAR071 HFSWR system. To overcome the angular resolution limit of the physical array, the multiple signal classification (MUSIC) algorithm is employe to achieve high-precision direction of arrival estimation. Additional radar parameters are summarized in Table 2.

### 3.3. Results of Simulated RFI

To analyze the impact of RFI mitigation on the sea-echo Doppler power spectrum, the Doppler extent of the simulated narrowband RFI is set from −0.35 Hz to −0.23 Hz, which spectrally overlaps with the negative Bragg peaks. The slow-time RFI signal is represented as a superposition of 40 discrete single-tone components with random amplitudes and initial phases, present across all sweeps. The average RFI power is −17.9 dB, with a spatial direction of arrival (DOA) fixed at 90°. To demonstrate the potential signal cancelation of the spatial OP within the main beam, a point target is simultaneously injected at the 30-th range bin (150 km) at a DOA of 90° and a Doppler frequency of −0.25 Hz. To clearly visualize the energy-spreading effect of the IPA waveform, the target SNR is set to 35 dB, which is 8 dB higher than the coherent integration gain ($10\log_{10}512\approx 27.1$ dB) provided by 512 sweeps.

**Figure 4:**
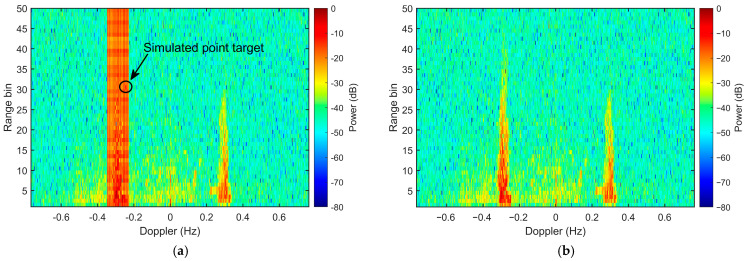
RDS of the field-measured sea echoes using the conventional waveform with injected simulated RFI and a point target. (**a**) Original RDS; (**b**) RDS after RFI mitigation via spatial orthogonal projection in the Doppler domain.

The RDS with superimposed simulated narrowband RFI and point targets is illustrated in Figure 4a. The noise floor is established at −44.0 dB, while the simulated point target at the 30th range cell has a power level of −9.0 dB. Although the intrinsic SNR of the target is high (35 dB), the masking effect of the intense RFI stripes leads to a mere 8.9 dB SINR at the target spectral bin. Such heavy interference masking significantly compromises target visibility, leading to potential missed detections in the raw RDS.

Figure 4b illustrates the RDS of the conventional waveform after RFI mitigation using the Doppler-domain spatial OP. Given that only the first 50 range cells of the range-time data are accessible, range cells 41 to 50 are designated for interference covariance matrix estimation. While the spatial OP in the Doppler domain effectively mitigates the RFI stripe, it simultaneously eliminates the point target at the 30-th range bin, as the target and interference share identical spatial and spectral characteristics.

**Figure 5:**
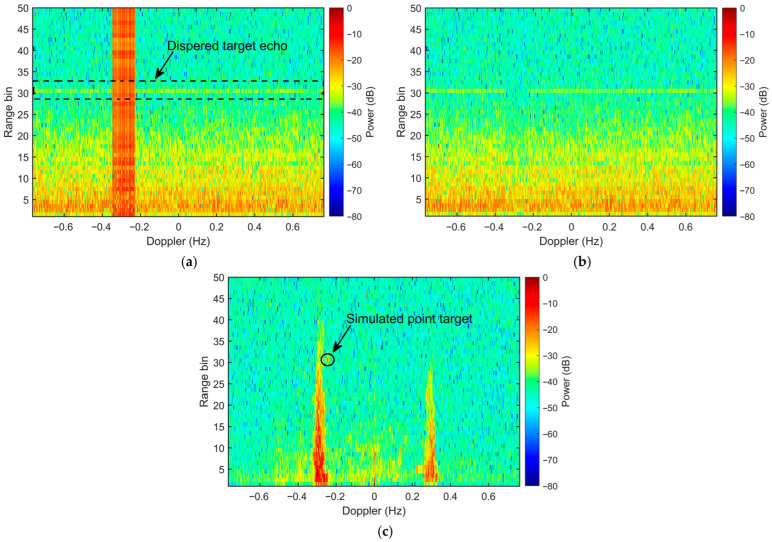
RDS of the field-measured sea echoes using the IPA waveform with injected simulated RFI and a point target. (**a**) Original RDS; (**b**) RDS after RFI mitigation via spatial orthogonal projection in the Doppler domain; (**c**) RDS after RFI mitigation and phase compensation.

In contrast, with the IPA waveform, the echo power of the desired target is dispersed across all Doppler bins within the 30-th range bin before phase compensation. This dispersion results in a horizontal band with a local SNR of approximately 8 dB (indicated by the dashed box in Figure 5a), representing the ‘whitened’ target signal. Similarly, the sea echoes are spread across all Doppler bins. Under these conditions, the RFI stripe only contaminates a small fraction of the desired signal samples—those falling within the specific Doppler range of the interference. Consequently, the majority of the target samples outside the RFI stripe remain unaffected.

After applying the spatial OP to all range-Doppler cells contaminated by the interference, the RFI is thoroughly suppressed (Figure 5b). During this process, the specific target samples overlapping with the interference stripe are inevitably eliminated, as their spatial signatures are identical to those of the RFI. However, target samples dispersed outside the interference Doppler bandwidth remain unaffected by the orthogonal projection. Following the phase compensation, these remaining samples regain their coherence, reconstructing a prominent target peak at the 30th range cell in Figure 5c.

**Figure 6:**
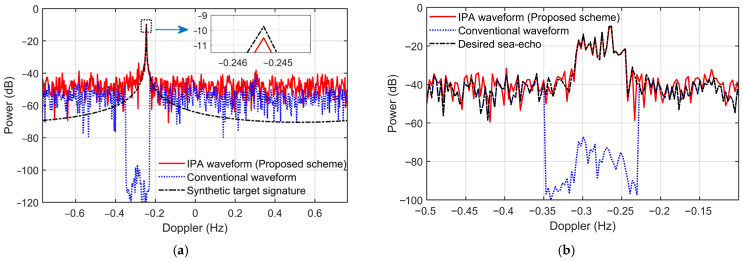
The beamformed Doppler spectrum after RFI mitigation with spatial projection in Doppler domain. (**a**) Results for the target range bin; (**b**) Negative Bragg region of the 3rd range bin.

Figure 6a shows the Doppler spectrum of the 30th range cell when the beam is steered toward the target DOA (90°). For the conventional waveform, the application of Doppler-domain spatial OP results in significant target self-cancelation. Consequently, the noise and clutter components at 90° are also nulled, leading to an artificially low Doppler floor within the processed frequency band. In contrast, the IPA waveform effectively preserves the target, with a power loss of only approximately 1 dB after phase compensation. It is observed that the ‘noise’ floor in the Doppler spectrum of the IPA waveform is slightly higher than that of the conventional waveform. The elevation of the ‘noise’ floor is attributed to spectral leakage caused by the signal discontinuity after interference excision, combined with the decoherence effect of the IPA. This ‘noise’ level is proportional to the target power and decreases as the PSL of the phase-agile sequence’s autocorrelation function is reduced.

Figure 6b illustrates the negative Bragg peaks of the sea echo at the 3rd range cell when the beam is steered toward the interference direction. Under conventional waveforms, the sea-echo signals within the RFI frequency bands are entirely suppressed. Conversely, with the IPA waveform, the power of the first-order Bragg peaks remains nearly constant before and after interference suppression.

The simulation results demonstrate that the IPA scheme enables the use of spatial filtering to mitigate RFI within the Range-Doppler domain, effectively addressing main-lobe interference with negligible loss to the desired signal. And the spectral distortion in the sea-echo Doppler spectrum induced by the proposed narrowband RFI suppression is significantly lower than that caused by conventional Doppler-domain spatial OP.

### 3.4. Results of Semi-Synthetic Data with Real-World RFI Overlays

In practical environments, the RFI signal is often highly complex, exhibiting time-varying parameters that fluctuate significantly across successive sweeps. Even strip-like narrowband RFI cannot be fully characterized by a simple superposition of finite single-frequency signals with constant amplitudes and initial phases. To rigorously verify the proposed scheme, we extract authentic narrowband RFI from raw experimental Doppler spectrum and superimpose it onto clean, interference-free measured data to create a semi-synthetic dataset. The experimental data were obtained from a multiple-input multiple-output (MIMO) HFSWR system optimized for ocean remote sensing. The system was deployed in Dongshan, Fujian, oriented toward the Taiwan Strait. The key radar operating parameters are summarized in Table 3.

**Figure 7:**
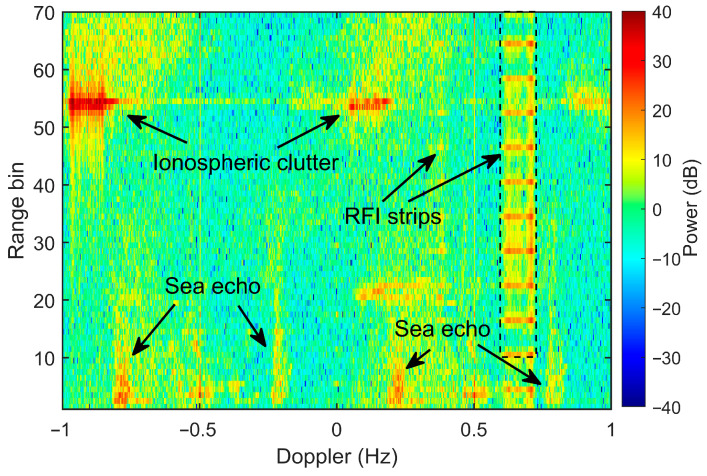
RDS of the interference-contaminated data collected on 31 December 2023, at 8:40 local time.

Figure 7 illustrates the RDS of the experimental data contaminated by field-measured RFI. The interference exhibits a distinct periodicity in the range dimension, with a cycle of approximately seven range bins. In this specific dataset, the RFI energy is primarily localized within the Doppler frequency band of 0.61 Hz to 0.72 Hz. Observations from late December 2023 reveal that while the RFI consistently appeared in the echo spectra, its Doppler morphology varied across different datasets. Despite these variations, the interference power remained localized within a narrow subset of Doppler bins. And in the slow time, the interference persisted throughout the entire CIT, maintained strong temporal coherence. In addition to the RFI, the RDS in Figure 7 displays two sets of sea echoes within the first 20 range bins. To achieve Doppler division multiplexing, these echoes are frequency-shifted to −0.5 Hz and 0.5 Hz. This ensures orthogonality between the two transmit channels and prevents the sea echoes from overlapping in the Doppler domain.

To prevent the presence of ship echoes within the near-range cells from biasing the assessment of sea-echo power deviation, interference-only samples are extracted from the 11th to 70th range bins of the dataset shown in Figure 7. These extracted RFI components are subsequently frequency-shifted and superimposed onto the 1st to 60th range bins of a ‘clean’ reference dataset.

**Figure 8:**
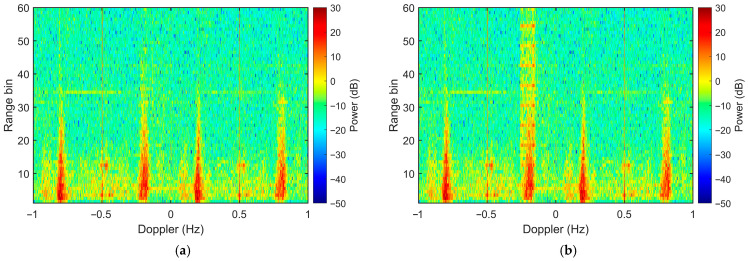
RDS of the semi-synthetic data. (**a**) Interference-free RDS derived from field-measured data with conventional waveform collected on 25 December 2023, at 2:45 local time; (**b**) Semi-synthetic RDS after superimposing real-world RFI extracted from the dataset in Figure 7.

The semi-synthetic RDS, generated by superimposing the real-world RFI extracted from Figure 7 onto the interference-free baseline (Figure 8a), is illustrated in Figure 8b. The injected RFI manifests as intense, range-extensive strips that effectively blanket the first-order Bragg peaks around −0.2 Hz.

**Figure 9:**
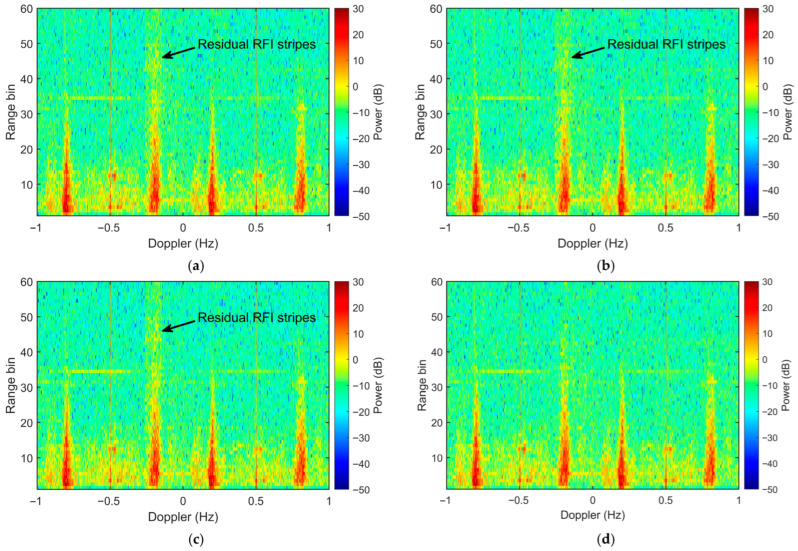
RDS of semi-synthetic data after interference mitigation with (**a**) Doppler-domain spatial OP, (**b**) frequency-dimension OP, (**c**) time-domain spatial OP and (**d**) proposed scheme (Doppler-domain spatial OP with IPA waveform).

For a comprehensive comparative analysis, three representative OP methods are implemented as benchmarks: the Doppler-domain spatial OP using conventional waveform, the frequency-dimension OP (utilizing frequency-dimension interference eigenvectors as proposed in [21]), and the time-domain spatial OP (proposed in [14]). All four OP-based algorithms utilize interference samples extracted from the 51st to 60th range bins to estimate the interference covariance matrix. As shown in Figure 9a–c, although all three benchmark methods suppress a substantial portion of the RFI power, they fail to achieve thorough mitigation, leaving discernible residual RFI strips in the RDS. In contrast, the proposed algorithm renders the residual RFI virtually undetectable (Figure 9d) and produces a pristine Range-Doppler map.

**Figure 10:**
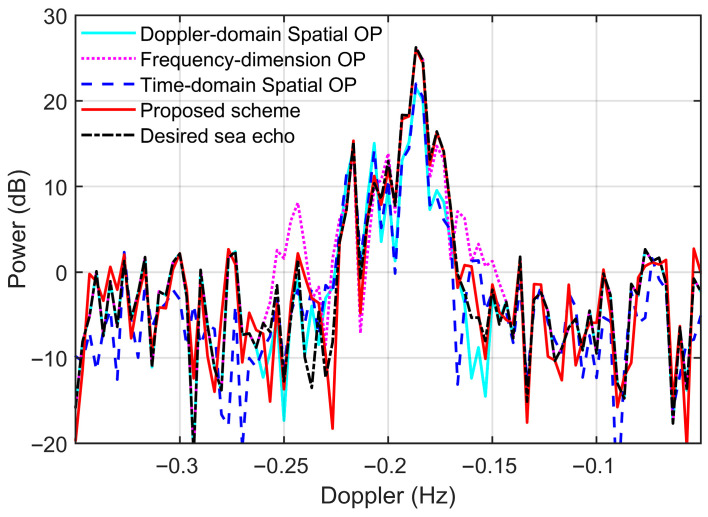
The beamformed Doppler spectra for the 6-th range bin after RFI mitigation.

Figure 10 provides a comparative analysis of the sea-echo Doppler power preservation for the approaching Bragg peaks (near −0.2 Hz) after RFI suppression. Since the impact of spatial OP is primarily concentrated within the main-lobe interference, the Doppler spectra shown here are obtained after conventional beamforming, with the main beam steered toward the RFI direction (estimated at 122° via conventional beamforming). As illustrated in Figure 10, both spatial OP algorithms utilizing the conventional waveform exhibit a noticeable reduction in the overall Bragg peak power. While the frequency-dimension OP method maintains negligible power deviation at high-SNR spectral bins, its performance degrades significantly in low-to-medium SNR regions—exemplified by the substantial power inflation observed around −0.25 Hz and −0.16 Hz. In contrast, the proposed IPA-based RFI mitigation scheme yields a Doppler spectrum that most closely aligns with the desired sea-echo Doppler spectrum, with discrepancies primary confined to low-SNR Doppler bins.

**Figure 11:**
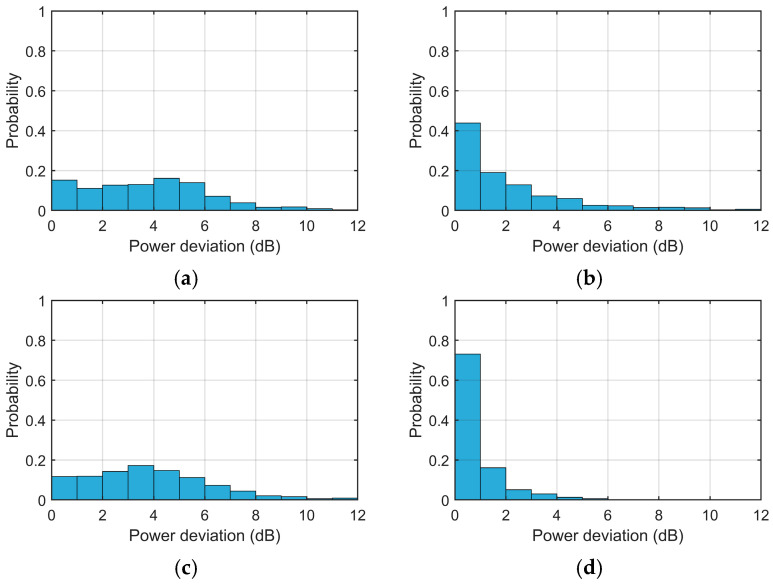
Statistical distribution of Doppler spectral power deviation in dB after RFI Suppression with (**a**) Doppler-domain spatial OP, (**b**) frequency-dimension OP, (**c**) time-domain spatial OP and (**d**) proposed scheme.

Figure 11 and Table 4 provide a statistical summary of sea-echo Doppler power deviations for spectral bins within the Bragg region (−0.26 Hz to −0.14 Hz) with an initial SNR exceeding 6 dB. The analysis is conducted on the beam-steered Doppler spectra with the main beam directed toward the interference source. The analysis reveals that the proposed IPA-based scheme achieves superior performance in signal preservation compared to the benchmarks. Specifically, the proposed method maintains a high level of spectral integrity, with over 89% of bins exhibiting deviations under 2 dB and an overall average deviation of only 0.9 dB. In contrast, the frequency-dimension OP method shows moderate performance with a 2.2 dB average deviation, while the two conventional spatial OP algorithms result in significant signal impairment. For these two spatial methods, less than 27% of the spectral bins maintained a deviation below 2 dB, with average power deviation about 4 dB. Results show that the proposed scheme effectively mitigates RFI while alleviating power distortion in the sea-echo Doppler spectrum.

## 4. Discussion

### 4.1. Discrepancy in Signal Attenuation Under Simulated RFI vs. Real-World RFI

In the simulation results of Figure 6b, the beam-steered Doppler spectrum after RFI suppression (blue dashed line), obtained via the Doppler-domain spatial OP, shows that the signal power within the interference frequency band is suppressed far below the noise floor. However, such extreme attenuation is not observed in the experimental results of Figure 10, where the actual power reduction remains within 12 dB (as statistically shown in Figure 11a). This discrepancy arises because the dominant eigenvector estimated from the real-world RFI does not exhibit strong spatial coherence with the theoretical steering vector of any specific direction. This phenomenon is likely attributable to DOA fluctuations of the RFI during the CIT or imperfect channel calibration at the interference azimuth.

### 4.2. Compatibility with DDM-MIMO Radar Architectures

In Section 3.4, real-world echoes from a DDM-MIMO HFSWR system are employed as idealized sea-echo signals for semi-synthetic data construction. Within this MIMO configuration, the echoes of the two transmit channels are frequency-shifted to −0.5 Hz and 0.5 Hz, respectively. This is achieved by modulating linear inter-pulse phase ramps to ensure waveform orthogonality in the Doppler domain. Although these linear phase ramps differ from the aperiodic sequences required by IPA waveform, the two techniques are inherently compatible. This is because the virtual aperture expansion provided by DDM-MIMO does not apply to externally generated RFI. Typically, RFI mitigation is performed prior to the separation of the multiple MIMO transmit channels. By superimposing the same aperiodic inter-pulse phase-agile sequence onto the original linear phase ramps of each transmit channel, the echoes from all channels will simultaneously undergo Doppler spreading and subsequent coherent recovery. Consequently, the proposed RFI mitigation scheme is fully applicable to DDM-MIMO radar configurations, maintaining its effectiveness without interfering with the MIMO waveform decoupling process.

### 4.3. Analysis of Algorithmic Generalization and Robustness

#### 4.3.1. Sensitivity to RFI Temporal Dynamics and Spectral Non-Stationarity

The proposed method operates within the Range-Doppler domain, necessitating that the RFI remains concentrated within a confined Doppler extent throughout the CIT. Consequently, the generalization of this approach is intrinsically linked to the temporal stationarity of the RFI. While dynamic RFI with sweep rates introduces spectral non-stationarity—leading to energy smearing and a subsequent drop in the localized INR—the algorithm demonstrates robust performance for the quasi-stationary interferences. Even for moderately non-stationary interference exhibiting limited spectral broadening, the proposed scheme remains effective. However, as the degree of spectral broadening intensifies, the attenuation of the desired signal during the suppression process increases accordingly. This trend, quantified by Equation (14), indicates that the algorithm’s advantage in protecting the target echoes gradually diminishes as the RFI energy redistributes. Ultimately, when the average power per Doppler bin within the RFI strip falls below the detection threshold due to extreme broadening, the algorithm reaches its performance boundary and may fail to identify the interference components.

#### 4.3.2. Robustness Against Spatial Signature Variations

Since the proposed method implements RFI suppression via spatial OP within the RDS, the stability of the interference’s spatial signatures throughout the CIT significantly dictates the algorithm’s performance. The most ideal scenario occurs when the RFI maintains a constant DOA, allowing the spatial OP to generate a singularly deep and narrow null. This ensures maximum interference cancelation with minimal impact on the surrounding spatial bins.

However, when the RFI experiences time-varying DOA shifts during the CIT, its representation in the RDS transitions from a localized point source to a distributed source with a finite angular spread. Due to the long integration time, components arriving from different paths and at different instances effectively lose their mutual coherence in the Doppler domain. Consequently, this temporal accumulation is physically equivalent to an incoherent distributed source. Assuming the RFI’s DOA varies linearly within an angular interval $\left[\theta_{0}-\Delta ,\;\theta_{0}+\Delta \right]$, it can be modeled as an incoherent source with a uniform angular power density. The resulting covariance matrix $R_{d}$ for such an incoherent distributed interference signal is given by:

(23) $$R_{d}(\theta_{0};\Delta )=\int_{\theta_{0}-\Delta }^{\theta_{0}+\Delta }\frac{1}{2\Delta }a\left(\beta \right)a^{H}\left(\beta \right)d\beta$$

where a(β) denotes the receiving array steering vector and Δ represents the angular spread of the interference signal. By incorporating the noise-perturbed covariance matrix of the distributed interference into the processing framework described in Section 2.4, the resulting distributed interference subspace, $U_{Jd}$, is derived. Consequently, the normalized spatial gain of the spatial OP, G(θ), can be expressed as:

(24) $$G(\theta )=\frac{{\left\|\left(I-U_{Jd}U_{Jd}^{H}\right)a\left(\theta \right)\right\|}^{2}}{a^{H}\left(\theta \right)a\left(\theta \right)}$$

**Figure 12:**
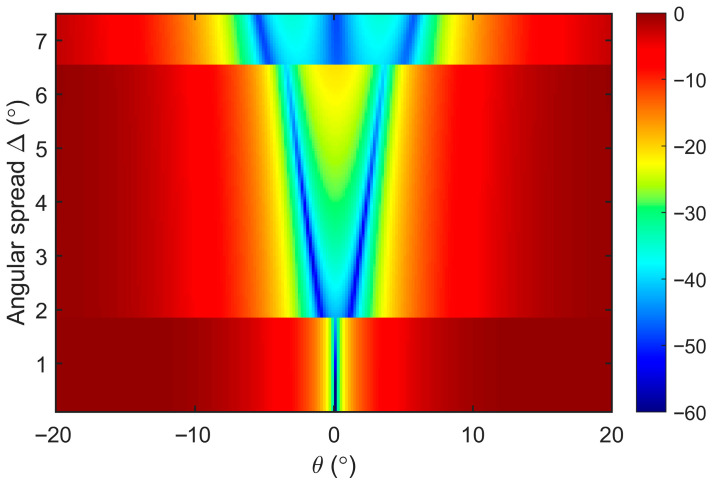
Normalized spatial gain of the spatial OP filter under varying degrees of RFI angular spread. The discontinuities observed in the plot mark the points where the estimated number of interference eigenvalues increases. As the angular spread increases, the number of dominant interference eigenvalues expands from one to three.

Figure 12 illustrates the normalized spatial gain of the spatial OP under varying degrees of RFI angular spread. The simulation employs an 8-element ULA with half-wavelength spacing, featuring a distributed interference centered at $\theta_{0}=0{}^{\circ}$ with an INR of 20 dB on the RDS. A total of 200 snapshots are utilized for the estimation of the noise-perturbed interference covariance matrix and subsequent subspace analysis. The results indicate that for an 8-element ULA, the performance of the proposed scheme remains highly robust as long as the interference angular spread is constrained within 1.8°, with the only marginal consequence being a noticeable shallowing of the spatial null depth produced by the OP operator. As the angular spread increases, the increasing number of interference eigenvalues estimated by the algorithm expands from one to three, causing a corresponding broadening of the spatial null generated by the OP.

Crucially, despite the widened spatial attenuation, the signal protection mechanism of the IPA waveform in the Doppler domain remains functional, demonstrating the method’s robustness against RFI with time-varying spatial signatures. However, as the angular extent further broadens or when multiple distributed RFI sources overlap in the RDS, the number of interference eigenvalues will continue to grow. Under such conditions, the limited degrees of freedom of the array will eventually impose a bottleneck on the algorithm’s applicability and suppression performance.

#### 4.3.3. Robustness Across Diverse Operational Frequencies

The performance of the proposed algorithm is primarily manifested in two aspects:

1. The preservation of desired signals facilitated by IPA-induced whitening and localized Doppler-domain processing.
2. The interference suppression efficacy achieved via spatial OP on the RDS.

The physical mechanism of IPA—which transforms desired echoes into a noise-like background prior to suppression—depends solely on the inter-pulse phase coding sequence and is frequency-invariant. Consequently, the signal protection performance is primarily governed by the Doppler occupancy of the interference, which in turn depends on its degree of non-stationarity.

At lower frequencies (<10 MHz), non-stationarity is dominated by ionospheric Doppler spreading, leading to noticeable spectral broadening in the RDS. Experimental results at 8 MHz in Section 3.3 demonstrate that the algorithm achieves excellent performance when the RFI occupies 14.4% of the total Doppler extent. According to Equation (14), which predicts a 3 dB signal power loss at 50% occupancy, the current results remain well within the operational margin. This suggests that the proposed method is robust against ionospheric-induced spectral broadening.

The intermediate HF band (10~20 MHz) represents the most congested region for shortwave broadcasting, where non-stationarity primarily arises from environmental complexity. Within this band, multiple interference sources with distinct DOAs are likely to coexist within a single CIT. If these sources overlap in the RDS, the estimated number of interference components (subspace rank) increases, necessitating a sufficient number of array elements to accurately estimate the interference subspace. Conversely, if the sources are spectrally separated in the RDS, they can be suppressed individually. However, the presence of multiple interference strips simultaneously increases the total Doppler occupancy, thereby compromising signal preservation performance.

In the higher HF band (>20 MHz), spectral broadening frequently originates from the intrinsic non-stationarity of the source, such as transient or frequency-hopping signals. These interferences are typically bursty and temporally discontinuous, resulting in severe spectral smearing that may occupy a large portion or even the entirety of the Doppler spectrum. In such scenarios, the algorithm’s performance declines due to two factors: (a) the drop in localized INR, which impedes RFI detection and accurate subspace estimation; and (b) increased signal attenuation caused by high spectral occupancy. Provided that the interference is successfully detected and its subspace is accurately estimated, the performance floor of the proposed method converges to that of classical adaptive beamforming, where signals within the main lobe suffer from severe self-cancelation.

#### 4.3.4. Robustness Against Varying Environmental and Ionospheric Conditions

While the proposed method demonstrates robust performance under typical conditions, weather-induced variations in the operational environment can influence the results in three primary aspects. First, sea-state conditions influence the propagation loss of surface waves, thereby modulating the received INR of surface-wave-propagated RFI. The proposed scheme demonstrates consistent robustness across diverse sea states by employing a dual-tier mitigation strategy: while high-intensity interference exceeding a predefined INR threshold is eliminated via spatial OP, sub-threshold components are effectively dispersed and whitened through the inherent phase-randomization properties of the IPA-based processing. Second, and more critically, ionospheric conditions fundamentally govern the non-stationarity and Doppler spreading of the RFI. Under turbulent ionospheric states, the increased spectral occupancy of the interference diminishes the signal protection efficacy, causing the performance to converge toward that of conventional spatial filtering. Finally, impulsive noise from thunderstorms spreads across the entire Doppler dimension, elevating the RDS noise floor. This may lead to low-intensity RFI components going undetected in the Doppler bins, thereby elevating the residual interference floor within the RDS after phase compensation. Such transient interference must be suppressed in the time domain as a pre-processing step before applying the proposed method.

## 5. Conclusions

This paper proposes a narrowband RFI mitigation scheme that synergistically combines inter-pulse phase agility waveform with Doppler-domain spatial orthogonal projection. By employing aperiodic inter-pulse phase-agile sequences with low autocorrelation sidelobes, the desired signals are uniformly randomized and spread across the entire Doppler spectrum prior to phase compensation, while the externally generated narrowband RFI remains coherently concentrated. Consequently, the spatial OP only suppresses a small fraction of the Doppler bins corrupted by RFI, thereby minimizing the loss of target signal energy. Validation using semi-synthetic data incorporating real-world RFI samples demonstrates that the proposed scheme effectively stabilizes the sea-echo Doppler spectrum, reducing the average power deviation of sea echoes from a range of 2.2–4.0 dB down to 0.9 dB. Furthermore, the proportion of spectral bins with a power deviation of less than 1 dB improved significantly from an initial range of 12–44% to 73.1%.

To further enhance the RFI suppression performance in HFSWR systems, our future research will focus on the following key areas:

- Field validation in complex environments: Deploying the proposed algorithms into operational radar systems to evaluate their robustness and real-time performance within complex, real-world electromagnetic environments.
- Robust RFI detection: Developing more stable RFI detection methods to ensure high reliability in adverse scenarios, such as severe ionospheric clutter or low-INR regime.
- Advanced source separation and signal isolation: Investigating sophisticated signal separation methodologies to achieve high-fidelity decoupling of RFI from desired echoes, moving beyond linear projection to better preserve the spectral integrity of the desired backscatter.
- Synergistic waveform agility: Integrating inter-pulse and intra-pulse agility to develop a robust suppression framework that leverages waveform diversity to mitigate non-stationary interference effectively.

## Figures and Tables

**Table 1:** Computational complexity of proposed scheme.

Stage	Operation	Complexity ^1^
Transformation	inverse FFT and FFT	$O\left(L\cdot M\cdot N\log N\right)$
Detection and SVD	SVD	$O\left(M^{3}\right)$
Suppression	Orthogonal projection	$O\left(N_{J}\cdot L\cdot M^{2}\right)$

^1^ Note: L denote the number of effective range bins.

**Table 2:** Experimental parameters of the OSMAR071 radar system.

Parameters	Value
Operating frequency	8 MHz
Average Transmitted Power	150 W
Radar waveform	FMICW (50% duty cycle)
Bandwidth	30 kHz
Sweep period	0.65 s
CIT	332.8 s (512 sweeps)
Doppler resolution	0.003 Hz
Receiving antenna array	x-axis: 27, 0, 18, 36, 54, 72, 90, 63 y-axis: −15, 0, 0, 0, 0, 0, 0, −15
Angular resolution ^1^	4.1°~7.6°

^1^ Note: The angular resolution is defined as the resolving power of the MUSIC algorithm under a reference condition of 20 dB SNR and 20 snapshots. The specified range covers a scanning sector from the boresight to 60° off-normal.

**Table 3:** Experimental parameters of the MIMO HFSWR system.

Parameters	Value
Operating frequency	8.15 MHz
Average transmitted power	300 W per channel
Radar waveform	FMICW (50% duty cycle)
Bandwidth	30 kHz
Sweep period	0.5 s
CIT	300 s
Doppler resolution	0.0033 Hz
Receiver array	8 monopole antennas (153 m total length)
Angular resolution ^1^	2.3°~4.6°
Transmitter configuration	2 antennas (142 m spacing)
MIMO strategy	Doppler division multiplexing

^1^ Note: The angular resolution is defined as the resolving power of the MUSIC algorithm under a reference condition of SNR = 20 dB and 20 snapshots. The specified range covers a scanning sector from the boresight to 60° off-normal. The potential aperture expansion provided by the MIMO configuration is not included in this baseline specification.

**Table 4:** Statistical comparison of power deviation for different RFI suppression schemes.

RFI Suppression Scheme	Bins with Deviation < 1 dB	Bins with Deviation < 2 dB	Average Power Deviation
Proposed IPA-based Scheme	73.1%	89.2%	0.9 dB
Frequency-dimension OP	43.8%	62.8%	2.2 dB
Doppler-domain spatial OP	15.2%	26.3%	4.0 dB
Time-domain spatial OP	11.7%	23.6%	4.1 dB

## Data Availability

The data presented in this study are available on request from the corresponding author.

## Abbreviations

The following abbreviations are used in this manuscript:

CIT	Coherent integration time
DOA	Direction of arrival
FFT	Fast Fourier transform
FMCW	Frequency modulated continuous wave
FMICW	Frequency modulated interrupted continuous wave
HF	High frequency
HFSWR	HF surface wave radar
INR	Interference-to-noise ratio
IPA	Inter-pulse phase agility
MIMO	Multiple input multiple output
MUSIC	Multiple signal classification
OP	Orthogonal projection
OSMAR	Ocean state monitoring and analyzing radar
PSL	Peak sidelobe level
RDS	Range Doppler spectrum
RFI	Radio frequency interference
SIR	Signal-to-interference ratio
SNR	Signal-to-noise ratio
SINR	Signal-to-interference-plus-noise ratio
SVD	Singular value decomposition
ULA	Uniform linear array
